# Cysteine and Aspartyl Proteases Contribute to Protein Digestion in the Gut of Freshwater Planaria

**DOI:** 10.1371/journal.pntd.0004893

**Published:** 2016-08-08

**Authors:** Louise S. Goupil, Sam L. Ivry, Ivy Hsieh, Brian M. Suzuki, Charles S. Craik, Anthony J. O’Donoghue, James H. McKerrow

**Affiliations:** 1 Department of Pharmaceutical Chemistry, University of California, San Francisco, San Francisco, California, United States of America; 2 Department of Pathology, University of California, San Francisco, San Francisco, California, United States of America; 3 Skaggs School of Pharmacy and Pharmaceutical Chemistry, University of California, San Diego, La Jolla, California, United States of America; Uniformed Services University, UNITED STATES

## Abstract

Proteases perform numerous vital functions in flatworms, many of which are likely to be conserved throughout the phylum *Platyhelminthes*. Within this phylum are several parasitic worms that are often poorly characterized due to their complex life-cycles and lack of responsiveness to genetic manipulation. The flatworm *Schmidtea mediterranea*, or planaria, is an ideal model organism to study the complex role of protein digestion due to its simple life cycle and amenability to techniques like RNA interference (RNAi). In this study, we were interested in deconvoluting the digestive protease system that exists in the planarian gut. To do this, we developed an alcohol-induced regurgitation technique to enrich for the gut enzymes in *S*. *mediterranea*. Using a panel of fluorescent substrates, we show that this treatment produces a sharp increase in proteolytic activity. These enzymes have broad yet diverse substrate specificity profiles. Proteomic analysis of the gut contents revealed the presence of cysteine and metallo-proteases. However, treatment with class-specific inhibitors showed that aspartyl and cysteine proteases are responsible for the majority of protein digestion. Specific RNAi knockdown of the cathepsin B-like cysteine protease (SmedCB) reduced protein degradation *in vivo*. Immunohistochemistry and whole-mount *in situ* hybridization (WISH) confirmed that the full-length and active forms of SmedCB are found in secretory cells surrounding the planaria intestinal lumen. Finally, we show that the knockdown of SmedCB reduces the speed of tissue regeneration. Defining the roles of proteases in planaria can provide insight to functions of conserved proteases in parasitic flatworms, potentially uncovering drug targets in parasites.

## Author Summary

Certain parasitic flatworms pose serious threats to human health. Understanding more about the biology of flatworms can lead to new and better drug targets. However, due to the complex life cycles of parasitic flatworms, it has been suggested that a non-parasitic flatworm, *Schmidtea mediterranea*, be used to study flatworm biology. This worm can be used as a model to study processes that all flatworms have in common; these findings can be applied back to parasitic flatworms. Digestion occurs in all worms and relies on the activity of protease enzymes to break down protein. The authors were able to induce regurgitation in *S*. *mediterranea* and study the contents of the worm gut. These worms have several types of proteases present, although it seems that only two types, called cysteine and aspartyl proteases, are important in digestion in live worms. Furthermore, one specific cysteine protease, cathepsin B, is responsible for the bulk of the digestive capabilities in *S*. *mediterranea*. Cysteine proteases also appear to be involved in the ability of *S*. *mediterranea* to regenerate damaged body tissues. Understanding protease activity in *S*. *mediterranea* can provide insight as to the roles of these enzymes in medically relevant parasitic flatworms.

## Introduction

The family *Platyhelminthes* contains an estimated 25,000 species, including the free-living planaria *Schmidtea mediterranea*. This freshwater flatworm has been an experimental model for over a century due to its regenerative capacity. Worms can fully regenerate missing body tissues from fragments as small as <1/200^th^ of their original size [1]. This is due to the large population of stem cells, or neoblasts, that make up approximately 30% of all adult tissues in the worm [2]. The *S*. *mediterranea* genome has been sequenced and these worms are highly amenable to the use of RNAi, making them an ideal flatworm for study [3,4].

While *S*. *mediterranea* is commonly used as a model for regeneration, it has recently been proposed as a model for parasitic flatworms, much like the free-living *Caenorhabditis elegans* is used as a model for other parasitic nematodes [5]. Over half of the known members of *Platyhelminthes* are human or veterinary parasites, including worms of the genus *Schistosoma*, the causative agents of the disease schistosomiasis. Schistosomiasis affects more than 200 million people worldwide and is the second most common parasitic disease behind malaria [6]. However, these and other parasitic worms are difficult to study due to their complex, multi-host life cycles and their resistance to biochemical tools such as RNA interference (RNAi).

*S*. *mediterranea* and the parasite *S*. *mansoni* share several similarities in their reproductive systems, protonephridia, and stem cell populations [7]. Both are triploblastic, bilaterally symmetric metazoans and contain nervous, digestive, and secretory systems [8]. In addition, these organisms share over 85% of their predicted proteome, including several families of proteases [9]. For digestion, *S*. *mansoni* and *S*. *mediterranea* have a blind-end, bifurcated gut that distributes digested food throughout the body [10]. Adult female *S*. *mansoni* worms will utilize several distinct proteases to rapidly digest red blood cells at a rate of 500 per minute [11]. While vertebrates rely on serine proteases from the trypsin family (clan PA) for protein digestion, invertebrate helminths generally use cysteine proteases from the clan CA (papain-like proteases) and aspartic proteases from the clan AA. Cysteine and aspartyl proteases are also key to digestion in other invertebrates like nematodes and arthropods. It appears that the preference for digestive serine proteases occurred during the evolution of arthropods or mollusks [12, 13, 14].

Three cysteine cathepsin proteases perform the majority of digestive function in helminths: cathepsins B, C, and L. These clan CA proteases are found in many flatworm parasites, including the trematodes *Fasciola hepatica*, *Clonorchis sinesis*, and *Opisthorchis viverrini* [11, 15]. Immunohistochemistry suggests that cathepsins B, C, and L are associated strongly with the gastrodermis, vitellaria, and tegument [15]. These proteases work in concert to degrade hemoglobin and albumin in the acidic helminth gut [16]. Inhibition of cysteine proteases has been shown to kill parasites both *in vitro* and *in vivo*, suggesting that these proteases are important for worm viability [17,16]. Previous work has shown that inhibition of schistosome cysteine cathepsin protease activity in infected mice has led to a reduced worm and egg burden and an improvement in organ pathology [18].

*Schistosoma* flatworms can be induced to regurgitate their gut contents and the cysteine proteases cathepsin B, C, and L, as well as an aspartyl protease (cathepsin D) and an asparaginyl endopeptidase (legumain), have been identified [19, 10]. Cathepsin B1, or SmCB1, represents the most abundant cysteine peptidase activity measurable in both adult schistosomes and gastrointestinal content (GIC) extracts. Along with degrading host hemoglobin and albumin, SmCB1 has been shown to degrade several immunoglobulins *in vitro*, suggesting possible roles in immune evasion [20]. Furthermore, SmCB1 has been suggested as a drug target, a potential vaccine target, and a serodiagnositc marker [21, 22]. Although schistosomula larvae with cathepsin B reduced by RNAi were still viable, these worms showed a significant decrease in growth compared to control groups [23]. This suggests that SmCB1 plays an important role in digestion such that a lack of activity has a negative effect on the acquisition of nutrients for growth.

Very little is known about the function of proteolytic enzymes in planaria and if conservation in enzymatic function exists between parasitic and free-living flatworms. Given the important role played by *S*. *mansoni* gut proteases in digestion, we investigated the role of these enzymes in the gut of *S*. *mediterranea*. Using proteomics and a global protease substrate profiling method, referred to as multiplex substrate profiling by mass spectrometry (MSP-MS), we identified and detected active proteases in *S*. *mediterranea* gastrointestinal contents. Using RNAi and specific protease inhibitors, we probed the function of several protease families *in vivo* to determine the roles of these enzymes in protein digestion. We further examined a cathepsin B-like cysteine protease and its localization in *S*. *mediterranea*. We hypothesized that planarians utilize cysteine proteases for digestion and that they perform similar roles in free-living flatworms as in parasitic helminths.

## Methods

### Colony care

A clonal line [4] of diploid, asexual *Schmidtea mediterranea* [24] was used in all experiments. Worms were maintained as described previously at room temperature (20–22°C) in 1x Montjuïc salts (worm water), pH 7.2, and were fed every two weeks with organic beef liver [25]. Unless otherwise stated, animals were starved for two weeks prior to use in experiments.

### Generation of worm regurgitant

Worms were starved for one week and washed several times in 1x Montjuïc salts before the addition of 3% EtOH for one hour to induce regurgitation. Treatment with low-percentage ethanol does not lead to long-term damage of worms [26]. Control samples were treated with water. Worm regurgitant was collected and filtered using a 50mm Filter Unit (Nalgene), then concentrated 50-fold using at an Amicon Ultra 10K MWCO Centrifugal Filter at 8,000 x *g* and 4°C. The concentrated vomit was stored at -80°C.

### Proteomic analysis of S. mediterranea regurgitant

Protein identification in *S*. *mediterranea* regurgitant was performed using peptide sequencing by mass spectrometry. 10μg total protein was digested with trypsin, extracted, desalted using C18 zip-tips (Rainin), and lyophilized. Liquid chromatography-tandem mass spectrometry was performed as previously described [27]. Algorithms in the BLAST2Go program (v2.7.2) were used to search for proteins with shared sequence features to proteins in *S*. *mediterranea* vomit. Searches were conducted against the SwissProt or NCBInr databases using the National Center for Biotechnology Information Server (July 27, 2015, blast.ncbi.nlm.nih.gov/Blast.cgi) and the top ranking hits reported in S1 Table.

### Protease assays

Protease activity in regurgitant from three replicate tanks were compared using a mixture of 7 internally quenched fluorescent substrates available from CPC Scientific, Sunnyvale, California (Table 1). These substrates were chosen based on their diverse sequence composition to enable detection of multiple protease classes. Worm regurgitant was diluted 7.5-fold in assay buffer containing 2.5 μM of each substrate, 100 mM NaCl, 2 mM DTT, 0.01% Tween-20 and 20 mM Citrate-Phosphate buffer at pH 3.5, 5.5 or 7.5. For inhibitor assays, the regurgitant from three replicate tanks were combined at equal volume and incubated with 10 μM Pepstatin-A, 10 μM E-64, 1 mM 1,10-Phenanthroline, 0.5 mM AEBSF or 1% DMSO. Assays were run for 1 hour at room temperature in black round bottom microplates using an excitation wavelength of 330 nm and emission wavelength of 400 nm. Activity was reported as change in fluorescent units per second.

**Table 1:** List of internally quenched fluorescent substrates used to detect cleavages in *S*. *mediterranea* regurgitant.

CPC Catalog #	Sequence
AMYD-112	Mca-His-Gln-Lys-Leu-Val-Phe-Phe-Ala-Lys(DNP)-NH2
AMYD-114	Mca-Glu-Val-Lys-Met-Asp-Ala-Glu-Phe-Lys(DNP)-NH2
AMYD-109	Mca-Ser-Glu-Val-Asn-Leu-Asp-Ala-Glu-Phe-Arg-Lys(DNP)-Arg-Arg-NH2
MMPS-024	Mca-Arg-Pro-Lys-Pro-Tyr-Ala-Nva-Trp-Met-Lys(DNP)-NH2
SUBS-017	Mca-Gly-Lys-Pro-Ile-Leu-Phe-Phe-Arg-Leu-Lys(DNP)-DArg-NH2
AMYD-111	Mca-Arg-Pro-Pro-Gly-Phe-Ser-Ala-Phe-Lys(DNP)
CASP-060	Mca-Val-Asp-Gln-Met-Asp-Gly-Trp-Lys-(DNP)-NH2

The MSP-MS assay was performed as previously described with minor modifications [28]. Worm regurgitant (500ng/mL) was assayed with an equal molar mixture of 124 tetradecapeptides (500nM each) in a total reaction volume of 300μL. Assays were performed at pH 3.5, 5.5, or 7.5 and incubated for 15, 60, 240, or 1200 minutes before quenching with concentrated formic acid to a final pH of 2.5. Samples were desalted with C18 zip-tips (Rainin) and analyzed by LC-MS/MS sequencing. Mass spectrometry and data analysis were performed as described previously [29]. To compare substrate specificity, iceLogo software (http://iomics.ugent.be/icelogoserver/) [30] was used to generate the specificity signature for amino acids at ±4 positions adjacent to the identified cleavage site.

### Identification of major cysteine proteases and cathepsin B in *S*. *mediterranea*

Sequences of *Schistosoma* proteases were obtained via BLAST and used to manually identify homologs found using the *Schmidtea mediterranea* database (SmedDb; http://planaria.neuro.utah.edu) [4]. Hits were checked via BLAST; reciprocal best hits were scored as putative homologs. The major cathepsin B homolog was isolated from planaria lysate (~50 planarians in lysis buffer; 100mM Tris pH 7.5, 200mM NaCl, 1% NP-40, 0.1% SDS, 1XTBS). Lysate was separated on a MonoQ 10/100 GL column (GE Healthcare Life Sciences) and assayed for protease activity with Z-RR-AMC (BACHEM). Fractions of cathepsin B activity were pooled and separated on a Superdex 200 10/300 GL column (GE Healthcare Life Sciences) which had been tested with a Gel Filtration Calibration Kit LMW (GE Healthcare) to determine at what volume the cathepsin B homolog would elute. The resulting samples with proteolytic activity were run on a 10% SDS-PAGE gel, silver stained, and cut into bands for analysis on PE-Biosystems Voyager Elite STR MALDI-TOF. The band corresponding to the most proteolytic activity and correct size was determined to be the cathepsin B homolog identified by SmedDb.

### Protease activity assays of whole worm lysates

To measure protease activity of worm lysates, planarians were ground with mortar and pestle for thirty seconds and incubated with lysis buffer (100mM Tris pH 7.5, 200mM NaCl, 1% NP-40, 0.1% SDS, 1XTBS) for one hour on ice with occasional vortexing. Samples were spun in a microcentrifuge at maximum speed, 4°C for twenty minutes. The supernatant was saved and activity was measured by adding assay mix (5mMDTT, 50mM sodium citrate, pH5.5) containing 50μM fluorescent peptide, either Z-FR-AMC or Z-RR-AMC (BACHEM). Fluorescence was measured (excitation 360nm, absorbance 460nm) using a FlexStation fluorometer (Molecular Devices) and SoftMax Pro 4.8 software. Both kinetic and endpoint assays were used. Chemical inhibitors of cathepsin B, K11777 (UCSF, CDIPD) and CA-074 (Sigma), were added at 50μM concentration two hours prior to addition of fluorescent peptides for select experiments. Protease activity was also measured with activity-based probes DCG-04 and BMV109, both gifts from Matthew Bogyo [31, 32]. Probes were added to 1μM final volume in samples containing 5mM DTT, pH 5.5 for 1 hour at 37°C before imaging Cy5 levels via Typhoon Trio (GE Healthcare Life Sciences).

### *In vivo* RNAi

*In vitro* double-stranded RNA (dsRNA) was synthesized from a PCR template of cathepsin B using T3 and T7 polymerases (Promega). The dsRNA was injected as described [33]. Worms were injected with three 33.2nL pulses of dsRNA (100ng total) once a day over three consecutive days. To determine the efficiency of knockdown, quantitative real-time PCR (qPCR) was performed using an Mx3005P QPCR System (Agilent Technologies) and LightCycler SYBR Green Master I (Roche). mRNA levels of cathepsin genes were compared to a standard internal control clone, GAPDH (Accession number: AY067285.1) to normalize RNA starting material. The efficiency and specificity of the primers was tested via serial dilutions of primers and cDNA template according to the protocol [34].

Primers used: GAPDH: 5’-AGCTCCATTGGCGAAAGTTA-3’, 5’- CTTTTGCTGCACCAGTTGAA -3’; CB1: 5’-CTAGATTCCAAACCGTTTCGGACA-3’, 5’-CAAGCTGCCAAAGAAAAGTTCAGG-3’; CL1: 5’-CAAGGCTATCCAGCAAATGG-3’, 5’-GAATCAACGCATTTGCAAT-3’; CD: 5’-GGCGAAATCACAATTGGAAC-3’, 5’-ATTTTCCATTCGATACGGCA-3’.

### Digestion and feeding assays

Planaria were fed pureed liver with tetramethylrhodamine conjugated bovine serum albumin (RhBSA, Molecular Probes). Worms were starved one week prior to feeding; RNAi treated worms were fed one week after injections were completed. Unless otherwise stated, worms treated with chemical inhibitors (K11777, pepstatin) received drug treatment for one hour prior to feeding. Worms were fed in the dark for one hour before being transferred into worm water (with or without appropriate drug) without food for one hour. Live worms were placed on slides on ice for a few minutes to stop worm movement before imaging. Images were taken using an Axiovert 40 CFL microscope and Axiovision Rel. 4.8.2 software (Zeiss). Fluorescence was quantified using ImageJ. Live worms were imaged again after 24 or 48 hours. Measurements were evaluated by paired t-tests; differences were considered significant with *P*<0.05.

### Antibodies

The catalytic region of *S*. *mediterranea* cathepsin B (based on the reference sequence mk4.000308.13.01) was cloned into pET28a and expressed in *Escherichia coli*. Protein was purified under reducing conditions (8M urea) on Ni-NTA Agarose (Qiagen) and dialyzed before mouse immunization. Sera was pooled and purified with NAb Protein G Spin Columns (Pierce). Three peptide regions of cathepsin B were selected and synthesized by New England Peptide, and combined to immunize rabbits by Covance. Antibodies were purified with NAb Protein G Spin Columns (Pierce).

### Whole-mount *in situ* hybridization

DNA template for *in vitro* transcription of anti-sense RNA probes was amplified from a PCR sequence containing the full length SmedCB1 gene. Probes were synthesized in an *in vitro* transcription with DIG-12-UTP (Roche) and precipitated with lithium chloride and ethanol according to the Roche suggested protocol. Probes were then resuspended and stored; animals between 2–5mm were processed for WISH as previously described [35] with modifications [36]. All animals were treated with 1:100 dilution of *SmedCB* probe.

### Immunofluorescence and electron microscopy

Animals were killed by treatment with 5% N-Acetyl L-Cysteine for five minutes at room temperature, fixed in 4% formaldehyde, and dehydrated in 50% MeOH. Planarians were embedded in paraffin, sliced, and treated with antibodies described above. For immunofluorescence, anti-rabbit and anti-mouse secondary antibodies with Alexa Fluor 488nm (LifeTechnonolgies) were used at 1:100. All imaging was performed using AxioImage.M1, Axiovision Rel. 4.8.2 software (Zeiss). Worms for electron microscopy were fixed and processed using the same procedures as the immunofluorescence. Worms were treated with the anti-zymogen antibody described above and an anti-rabbit 10nm gold preadsorbed secondary antibody (AbCam).

### Regenerative assays

To measure the effect of cathepsin B RNAi on regeneration, planarians were amputated one full day after injections were complete. Worms treated with cathepsin B inhibitors were also amputated pre- and post-pharyngeally and immediately placed in worm water containing the appropriate drug. Unless otherwise indicated, images for size quantitation were taken immediately after amputation (Day 0) and again on days 5, 8, 11, and 14. Worms saved for RNA extraction for qPCR were also frozen at -80°C on these days. Live images were taken using an AxioZoom.V16 microscope and Axiovision Rel. 4.8.2 software (Zeiss). Phenotypes were scored by measuring body areas with ImageJ. Each planaria amputated fragment (head, pharynx, and tail) was measured for change in total area and blastema size. Changes in area were evaluated with paired t-tests; differences were considered significant with *P*<0.05.

## Results

### Several protease families are conserved between *S*. *mediterranea* and the parasite *S*. *mansoni*

A preliminary BLAST search using sequences from previously identified *S*. *mansoni* proteases against the *S*. *mediterranea* database (http://smedgd.neuro.utah.edu) revealed that several protease families are conserved between both parasitic and free-living flatworms (Table 2). Among the conserved protease families were aspartyl and cysteine cathepsin proteases, including cathepsin B, D, L1, and L2/L3, all of which are involved in blood feeding and found in the gut of in *Schistosoma* worms. The aspartyl proteases cathepsin D had two putative homologs, while the cysteine protease cathepsin L1 and L2/L3 each had several putative homologs. Unlike *S*. *mansoni*, only one gene corresponding to cathepsin B was found in *S*. *mediterranea*. Interestingly, two well-characterized *S*. *mansoni* proteases were absent in the *S*. *mediterranea* genome. These enzymes are the serine protease, cercarial elastase, which is involved in skin invasion, and the gut-associated asparaginyl endopeptidase called legumain [10].

**Table 2:** Homologous *Schistosoma mansoni* proteases found in *Schmidtea mediterranea*. BLAST comparisons of known *S*. *mansoni* proteases with the *S*. *mediterranea* genome database (http://smedgd.neuro.utah.edu/). Some proteases, like those in the cathepsin (Clan CA, papain) family, were conserved with 1 or more homologs. As expected, the cercerial protease used by schistosomes for skin invasion is absent. Surprisingly, there appears to be no legumain (SmAE) homolog, which is found in many helminths, including *S*. *mansoni*.

Protease	Present in *Schimdtea*	Number of Homologs	Function in *S*. *mansoni*	Localization in *S*. *mansoni*
**Cathepsin B1**	Yes	1	Blood feeding	Gut (cecum)
**Cathepsin B2**	Yes	1	Unknown	Tegument
**Cathepsin D**	Yes	2	Blood feeding	Gut
**Cathepsin L1 (F)**	Yes	3	Blood feeding	Gut
**Cathepsin L2**	Yes	17	Blood feeding	Gut
**Cathepsin L3**	Yes	20	Blood feeding	Gut
**Leucine Aminopeptidase (1&2)**	Yes	5	Hatching and unknown gut function	Gastrodermis
**Kallikrein**	Yes	2	Unknown; suggested to be modulation/evasion of host immune system	Schistosomula released products and male dorsal tubercles
**M8 (Invadolysin)**	Yes	8	Unknown	Acetabular glands
**Legumain (SmAE, asparaginyl endopeptidase)**	No	0	Unknown; suggested to be hemoglobin degradation	Gut (cecum)
**SmCE (cercarial elastase)**	No	0	Invasion through skin	Acetabular glands and pre-acetabular glands

### Cysteine proteases represent the major proteolytic activity in *S*. *mediterranea* and its gut

**Fig 1:**
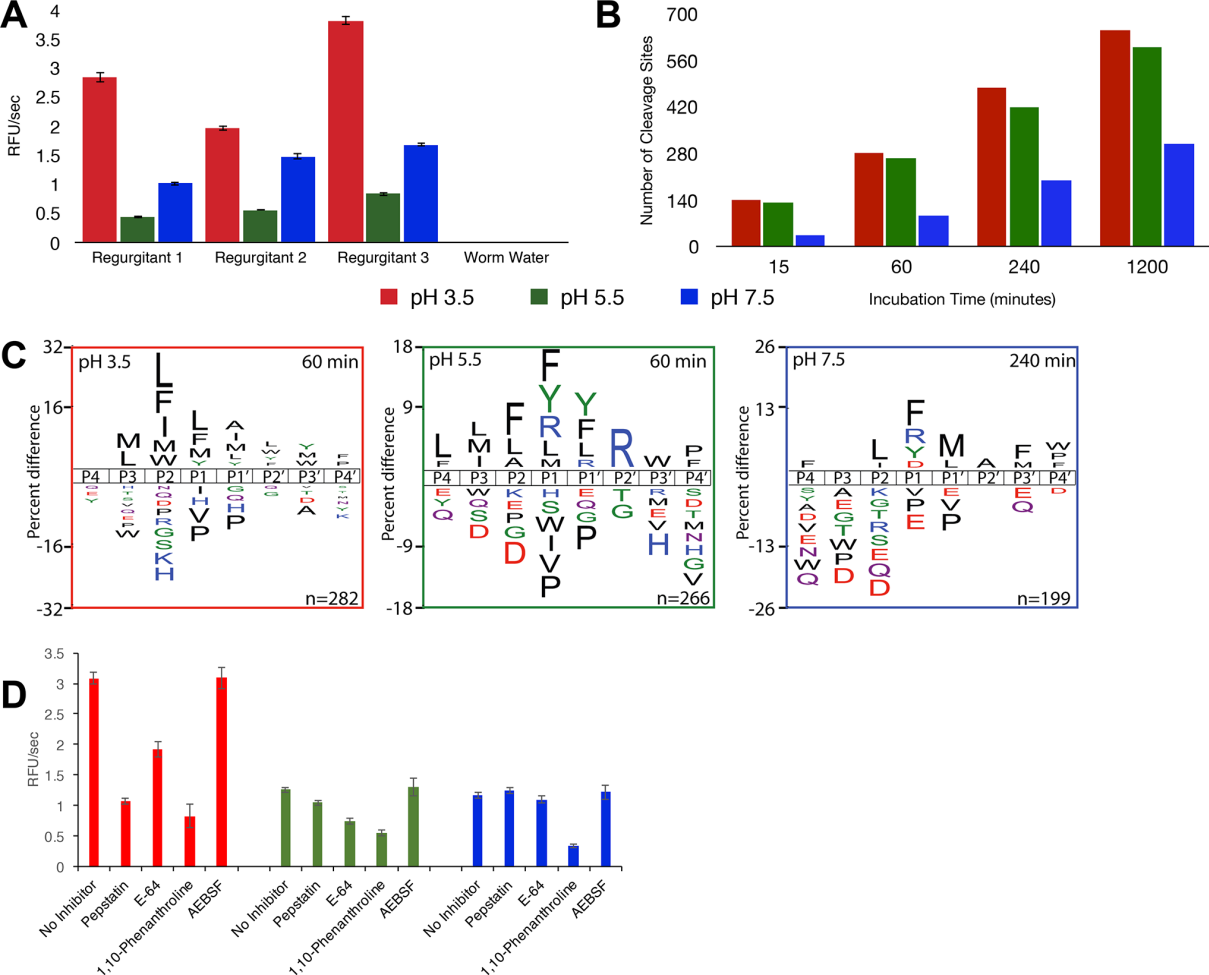
Incubation of worm regurgitant with fluorescent substrates and MSP-MS reveals highest activity at low pH and is dependent on aspartyl, cysteine, and metalloproteases. **(A)** Replicates of *S*. *mediterranea* regurgitant were incubated with a library of fluorescent substrates at pH 3.5, 5.5, and 7.5. Each sample showed the highest rate of cleavage at pH 3.5. No detectable activity was found in samples without regurgitant. Fluorescence was measured in relative fluorescence units (RFU) per second. **(B)** The regurgitant was incubated with peptide substrates for MSP-MS and the total number of cleavage sites was detected after 15, 60, 240, and 1200 minutes of incubation with the peptide mixture at pH 3.5, 5.5, and 7.5. The largest number of cleavages occurred at pH 3.5. **(C)** An iceLogo generated from the pattern of cleavage events after 60 minutes reveals the specificity of protease activity at pH 3.5, 5.5, and 7.5. Amino acids that are most frequently observed at each position (P4-P4’) are shown above the axis, while less frequently observed amino acids are shown below. **(D)** The pooled samples of regurgitant were assayed with the same fluorescent library in the presence of several inhibitors, including pepstatin (aspartyl protease inhibitor), E-64 (cysteine protease inhibitor), 1,10-phenanthroline (metalloprotease inhibitor), and AEBSF (serine protease inhibitor). While the effect of inhibition of astpartyl, cysteine, and metalloproteases varied according to pH, inhibition of serine proteases had no effect on the overall amount of activity detected at any pH.

To identify and characterize intestinal tract proteases in *S*. *mediterranea*, worms were placed in 3% ethanol to induce regurgitation (S1 Video). These worms were starved for a week to avoid contamination by proteases from the *S*. *mediterranea* food source. Protease activity in the worm regurgitant was assessed using a mixture of internally quenched fluorescent substrates. These substrates consist of 7-mer to 10-mer peptide sequences flanked by 7-methoxycoumarin on the amino terminus and dinitrophenol conjugated to lysine on the carboxyl terminus. Protease activity from replicate *S*. *mediterranea* tanks was assayed in pH 3.5, 5.5 and 7.5 buffer. Activity was highest at pH 3.5 and lowest at pH 7.5. No activity was detected in the water from worms that were not exposed to ethanol (Fig 1A). The increase in protease activity due to ethanol treatment proved that we had successfully enriched for the gut proteases of *S*. *mediterranea*. The regurgitant from the replicate tanks was pooled and the substrate specificity profile was uncovered using an unbiased substrate profiling assay consisting of 124 physiochemically diverse peptides of 14 residues each [28]. Cleavage of these peptides was detected by LC-MS/MS sequencing after incubation of regurgitant with the peptide mixture at pH 3.5, 5.5 and 7.5. An aliquot of each reaction was removed after 15, 60, 240, and 1200 minutes. Proteases active at pH 3.5 and 5.5 cleaved at more sites than the proteases that were active in the pH 7.5, indicating a broader substrate specificity (Fig 1B). Proteases active in the pH 3.5 buffer had a preference for all hydrophobic residues except Val and Pro at most positions between P3 and P4ʹ (Fig 1C). In the pH 5.5 buffer, hydrophobic residues were also found at high frequency in most sub-sites, except for P2ʹ, which prefers Arg. In addition, Tyr and Arg are well tolerated at P1 and P1ʹ. Unlike the cleavage sites detected in the pH 3.5 and 5.5 assays, protease activity at pH 7.5 was dominated by exo-peptidases. After 15 minutes incubation, 30 out of 36 cleavage sites detected occurred at the amino or carboxyl termini of the 14-mer peptides. After 60 minutes incubation, 68 out of the 94 cleavage sites had occurred at the termini and the overall substrate preference was for arginine at P1 and norleucine at P1ʹ. Taken together, these data indicate that multiple proteases are present in the gut of *S*. *mediterranea* that are capable of cleaving diverse set of peptide bonds over a broad pH range.

In order to better understand the proteases responsible for cleaving the peptide substrates, we incubated *S*. *mediterranea* regurgitant with the class specific protease inhibitors pepstatin-A, E-64, 1,10-phenanthroline, and AEBSF. These compounds are standard inhibitors of aspartyl, cysteine, metallo-, and serine proteases, respectively. Under all conditions tested, AEBSF failed to inhibit activity, indicating that there were no active serine proteases present in the regurgitate (Fig 1D). At each pH, 1,10-phenanthroline reduced activity by more than 50%, while pepstatin and E-64 inhibited protease activity at pH 3.5 and pH 5.5 only.

To identify the specific proteins responsible for the aspartyl-, cysteine- and metallo-protease activity in the *S*. *mediterranea* regurgitant, we performed a proteomic analysis on the regurgitant proteins. Proteins were digested with trypsin, and the resulting peptides were sequenced with liquid chromatography-tandem mass spectrometry (LC-MS/MS). These peptides were compared to a database of putative proteins from the asexual *S*. *mediterranea* strain (http://smedgd.stowers.org/downloads/). Peptides were ranked based on their intensity sums. We identified a total of 122 proteins, including 7 proteases (S1 Table). Based on peptide count, the two metalloproteases, astacin-2 and astacin-5 appear to be the most abundant proteases in the regurgitant. Recombinant astacin from the parasitic nematode, *Teladorsagia circumcincta* was expressed in *E*. *coli* and found to be sensitive to 1,10-phenanthroline [37]. Therefore, it is likely that astacin-2 and -5 are the source of the 1,10-phenanthroline-sensitive activity in *S*. *mediterranea* regurgitant. We also found that one regurgitant sample contained three other metalloproteases: an M12 protease, astacin 1, and two M10 matrix metalloproteinases, mmp1 and mmp2. Although our inhibition studies determined that there was one or more pepstatin-sensitive aspartyl protease in the gut, we were unable to identify this protease by mass spectrometry; it is possible that aspartyl proteases are in lower abundance than metallo- or cysteine proteases. However, we were able to detect two clan CA cysteine proteases; a cathepsin B-like and a cathepsin L1 protease that are likely to be the E-64 sensitive enzymes in the pH 5.5 assay.

### Inhibition of cysteine and aspartyl proteases, but not metalloproteases, blocks digestion in *S*. *mediterranea*

**Fig 2:**
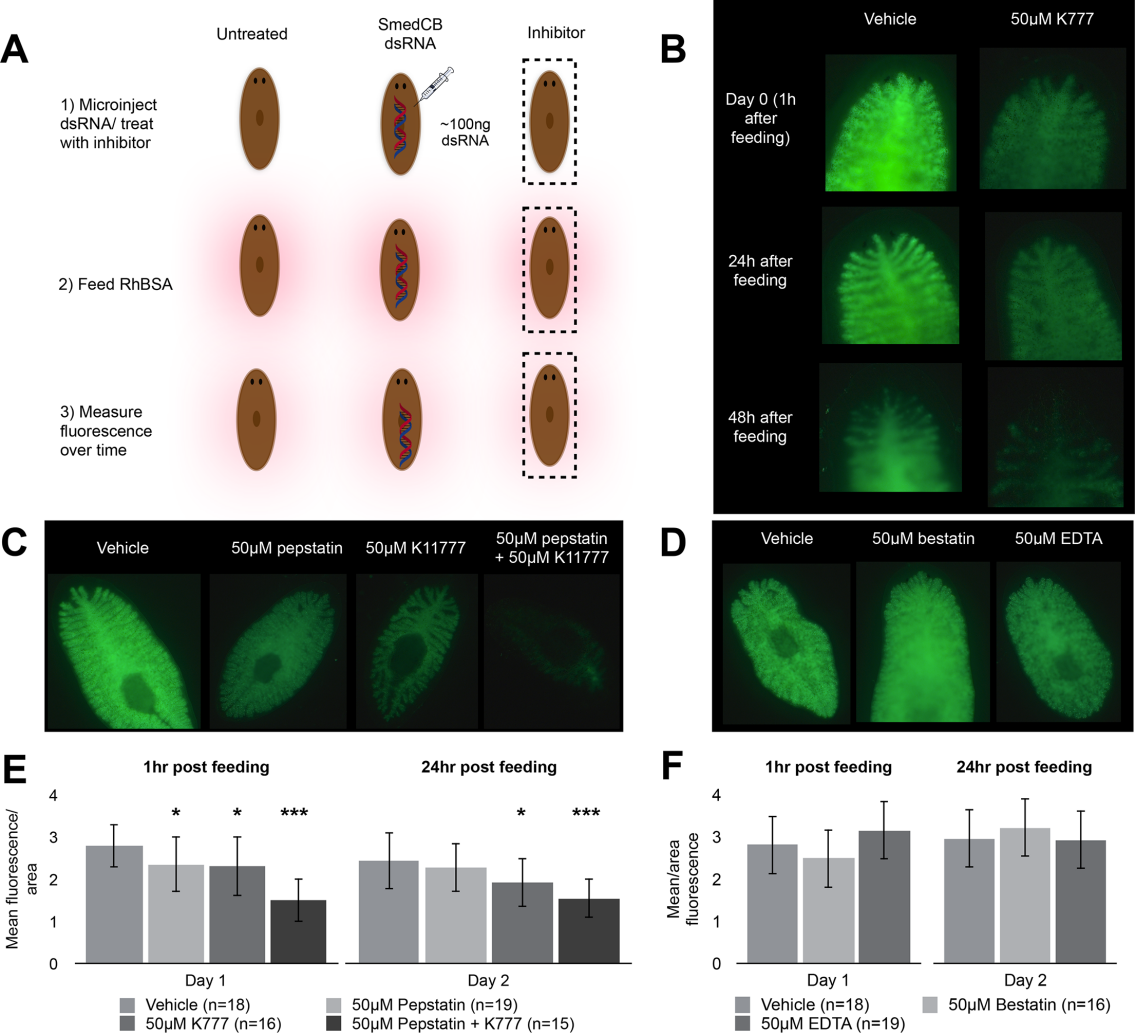
Inhibition of cysteine, aspartyl, but not metalloproteases, inhibits planaria digestion. **(A)** Animals were treated with 50μM protease-specific chemical inhibitors or dsRNA prior to feeding with rhodamine-labeled bovine serum albumin (RhBSA) and imaging. Fluorescence, due to cleavage of quenched albumin, was imaged **(B).** These representative images show a decrease in fluorescence in worms treated with K11777. Inhibition of aspartyl proteases by pepstatin as seen in **(C)** also decreased digestion. Combined inhibition of cathepsin B, L, and D completely reduced fluorescence. In contrast, inhibition of metalloproteases with bestatin and EDTA **(D)** did not reduce fluorescence. These data are quantified in **(E)** for K11777 and pepstatin and show that the reduction in fluorescence is significant in the presence of these inhibitors. **(F)** Treatment with metalloprotease inhibitors bestatin and EDTA did not lead to a significant change in fluorescence.

As noted above, cysteine, aspartyl, and metalloproteases are all active in the regurgitant of *S*. *mediterranea* and are all likely to play important yet distinct roles in protein digestion. To investigate the multi-enzyme model of digestion, we utilized a chemical knockdown strategy using protease inhibitors to monitor the effect of these protease classes. Starved worms were fed beef liver coated with rhodamine-labeled bovine serum albumin (Rh-BSA), which has been used previously to assay digestive proteases in *S*. *mansoni* [10]. Fluorescence generated by cleavage of quenched rhodamine-BSA molecules is proportional to the activity of digestive enzymes; lower fluorescence indicates a decrease in digestion (Fig 2A). Measurements are represented as fluorescence over worm area to correct for differences in worm size.

We first examined the role of cysteine proteases, specifically the two identified in the worm regurgitant: cathepsin B and cathepsin L. While E-64 is the standard pan-cysteine protease inhibitor *in vitro*, its poor cell-permeability limits its effects *in vivo* [38]. However, the vinyl sulfone K11777 is a potent chemical inhibitor of both cysteine proteases cathepsin B and L, is cell-permeable, and has been shown to reduce the parasite burden of schistosomes in mice [39, 18]. Chemical inhibition of cysteine cathepsin proteases by K11777 strongly reduced digestion in the planaria gut. Worms pretreated with K11777 were fed RhBSA and fluorescence was imaged as a measurement of proteolytic activity. K11777 treated worms exhibited a 38% reduction in fluorescence compared to untreated controls (Fig 2B and 2E). This decrease in signal persisted over time, and twenty-four hours post feeding treated worms had a 42% decrease in signal.

As one or more pepstatin-sensitive aspartic proteases are present in the *S*. *mediterranea* regurgitant, we used this inhibitor to determine the role of these enzymes in protein digestion. Worms treated with pepstatin resulted in a 16% reduction in RhBSA activity immediately after feeding compared to the untreated worms. After 24 hours, only a 6% decrease in activity was evident. However, co-treatment with pepstatin and K11777 had a dramatic effect on digestion. Treated worms were on average 46% and 37% less bright than vehicle treated worms after one and twenty-four hours, respectively (Fig 2C and 2F). The representative image in Fig 2C shows that very little fluorescence, indicative of albumin degradation, was observed in worms where both cysteine and aspartic proteases were inhibited.

Due to the presence of metalloproteases in the worm regurgitant as documented by biochemical assays and the proteome analysis, 1,10-phenathroline was used to examine the effect of reducing the metalloprotease activity. However, 1,10-phenanthroline was highly toxic to the worms and therefore two alternative metalloprotease inhibitors, bestatin and EDTA, were used for the *in vivo* digestion studies. Representative images in Fig 2D show that no decrease in fluorescence due to albumin degradation was detected; therefore, digestion of rhodamine-labeled albumin was unaffected by metalloprotease inhibitors (Fig 2D and 2F).

### Knockdown of SmedCB inhibits digestion

**Fig 3:**
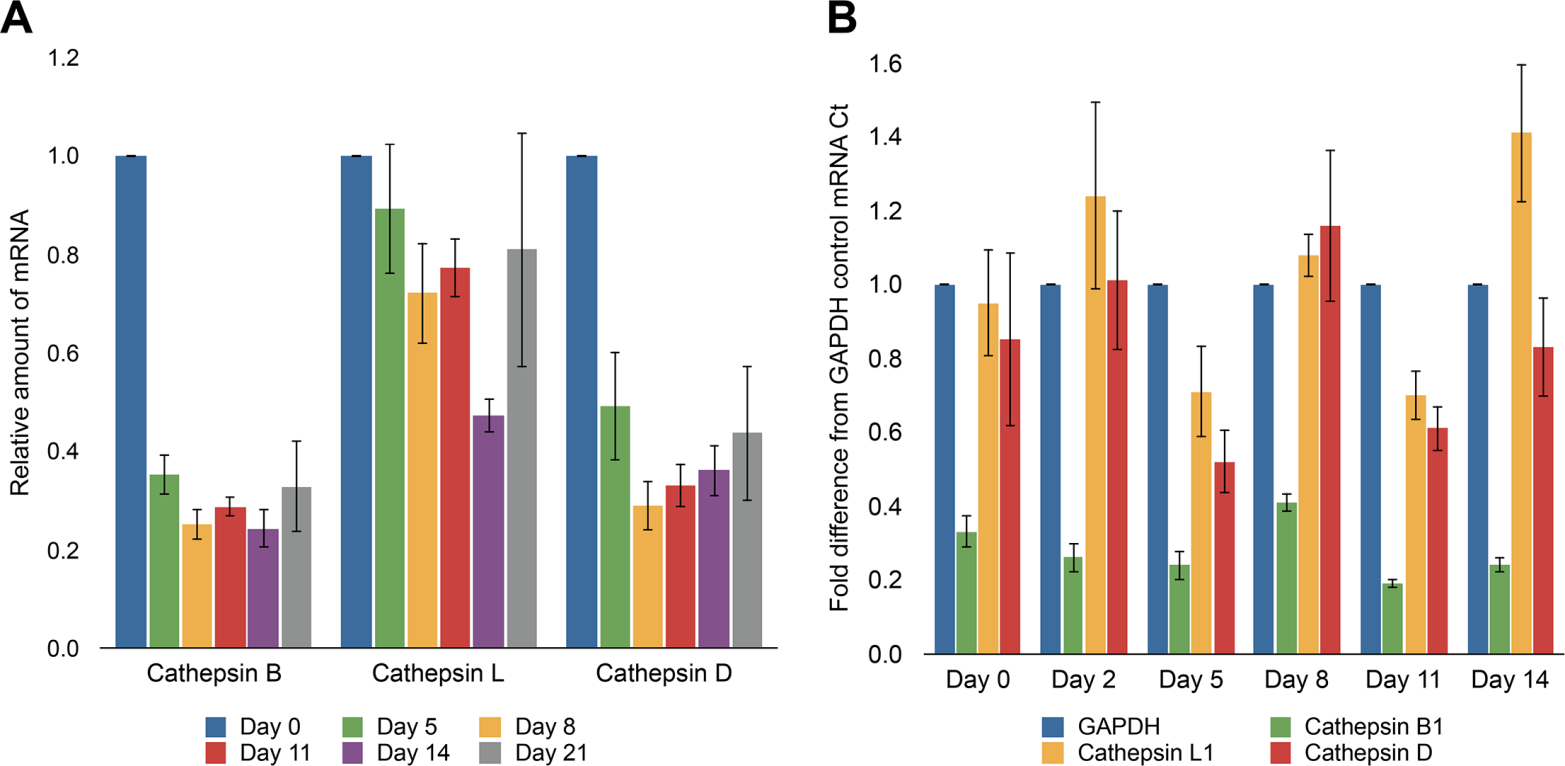
*S*. *mediterranea* cathepsin protease mRNA is reduced after starvation while dsRNA treatment with SmedCB is specific to cathepsin B. **(A)** RNA was extracted from three sets of 10 to 15 worms starved for three weeks. mRNA levels for three major cathepsin protease genes (B, L, and D) were quantified using RTPCR; levels were compared to recently fed worms (day 0) to measure the relative change in transcript levels over time. Starved worms demonstrated a 65–75% reduction in cathepsin B mRNA levels and 50–65% reduction in cathepsin D mRNA. Cathepsin L mRNA was reduced, on average, 20% with the exception of day 14, which had a 50% reduction. **(B)** Worms injected with ~100ng of SmedCB dsRNA for three consecutive days, followed by amputation (day 0) and three weeks of starvation, showed a marked reduction of SmedCB mRNA when analyzed via RTPCR using GAPDH mRNA levels as a baseline control. SmedCB levels decreased by ~80% after two weeks. Cathepsin L and D showed some fluctuation in mRNA levels, but remained within 50% of baseline levels and there was no continued trend observed over time. This indicates that fluctuations in levels are due to variations in worm populations chosen for RNA extraction rather than significant off-target effects of SmedCB dsRNA.

The reduction in protein digestion in *S*. *mediterranea* that occurs in the presence of a combined treatment of a cysteine and aspartyl protease inhibitor confirms that these proteases are the major digestive enzymes in the worm gut. Although we could not confirm the exact aspartyl protease present, we predicted that this enzyme was a cathepsin D-like protein found in the genome of planaria. Primer pairs were designed against the mRNA sequence of cathepsin D and the two cysteine proteases, cathepsin B and L. We first used RT-PCR to quantify the levels of cysteine and aspartyl proteases over time following starvation. Worms starved over a three-week period showed a 50–65% reduction in cathepsin D levels and a 65–75% reduction in cathepsin B mRNA levels while cathepsin L levels were reduced by only 20% (Fig 3A). These data suggest that cathepsin B and D expression is regulated by food intake while cathepsin L expression is less affected by changes in feeding conditions.

**Fig 4:**
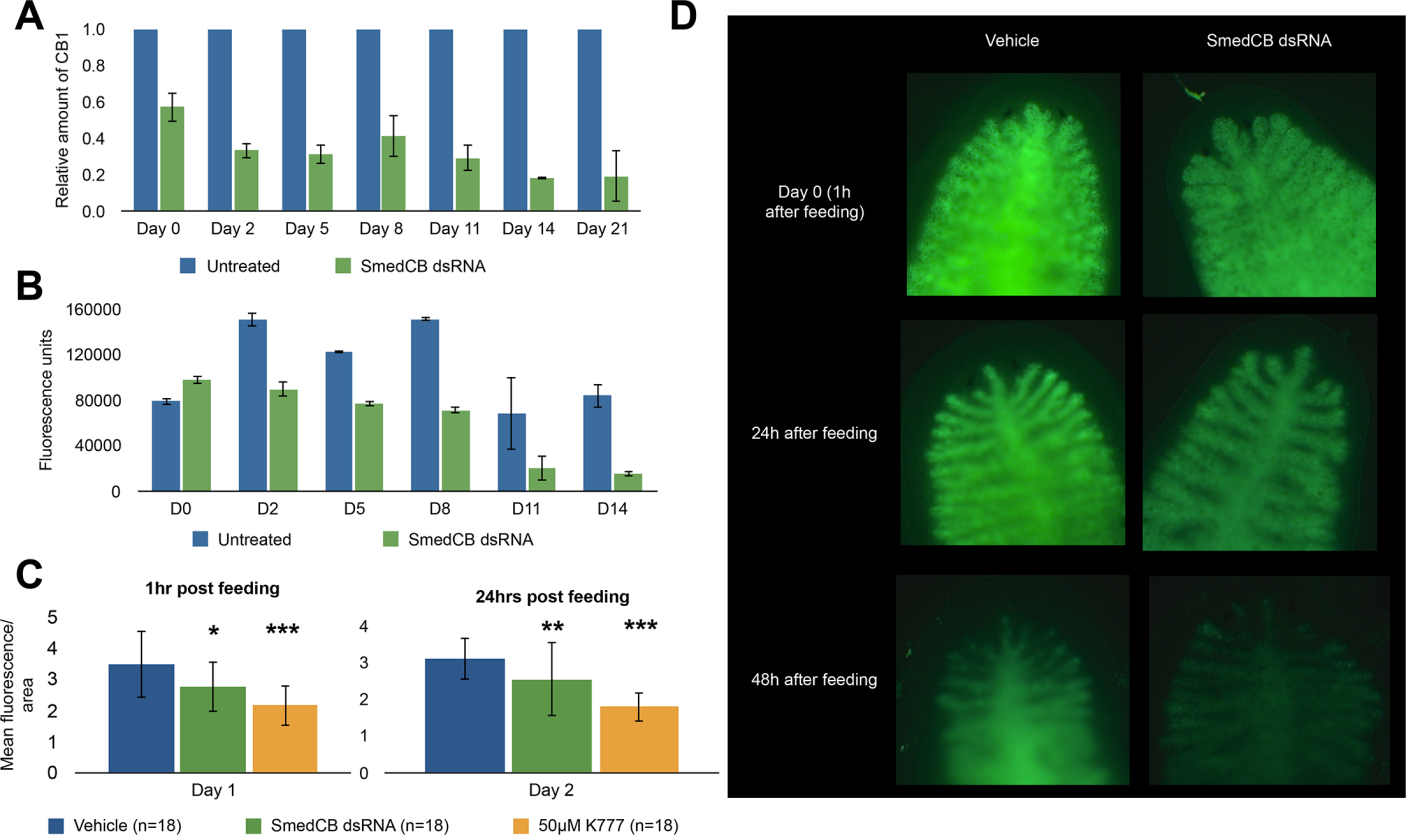
SmedCB RNAi treatment reduces cathepsin B mRNA levels and proteolytic activity, inhibiting digestion. **A)** Worms injected with ~100ng of SmedCB dsRNA for three consecutive days, followed by amputation (day 0) and three weeks of starvation, showed a marked reduction of SmedCB mRNA when analyzed via RTPCR in comparison to untreated worms. The initial three days of injection prior to day 0 causes a 45% reduction that increases to over 80% after two weeks of starvation during regeneration. SmedCB dsRNA treated worms also showed a marked reduction of SmedCB protease activity via Z-RR-AMC cleavage **(B)**. Treated worms exhibited between 50–75% less fluorescence from Z-RR-AMC cleavage than untreated worms over two weeks. **C)** Fluorescence from digestion of Rh-BSA is decreased in worms treated with SmedCB dsRNA. This decrease is less dramatic than treatment with the cathepsin B and cathepsin L inhibitor K11777, but is statistically significant. Representative images of this fluorescence can be observed in **D**.

**Fig 5:**
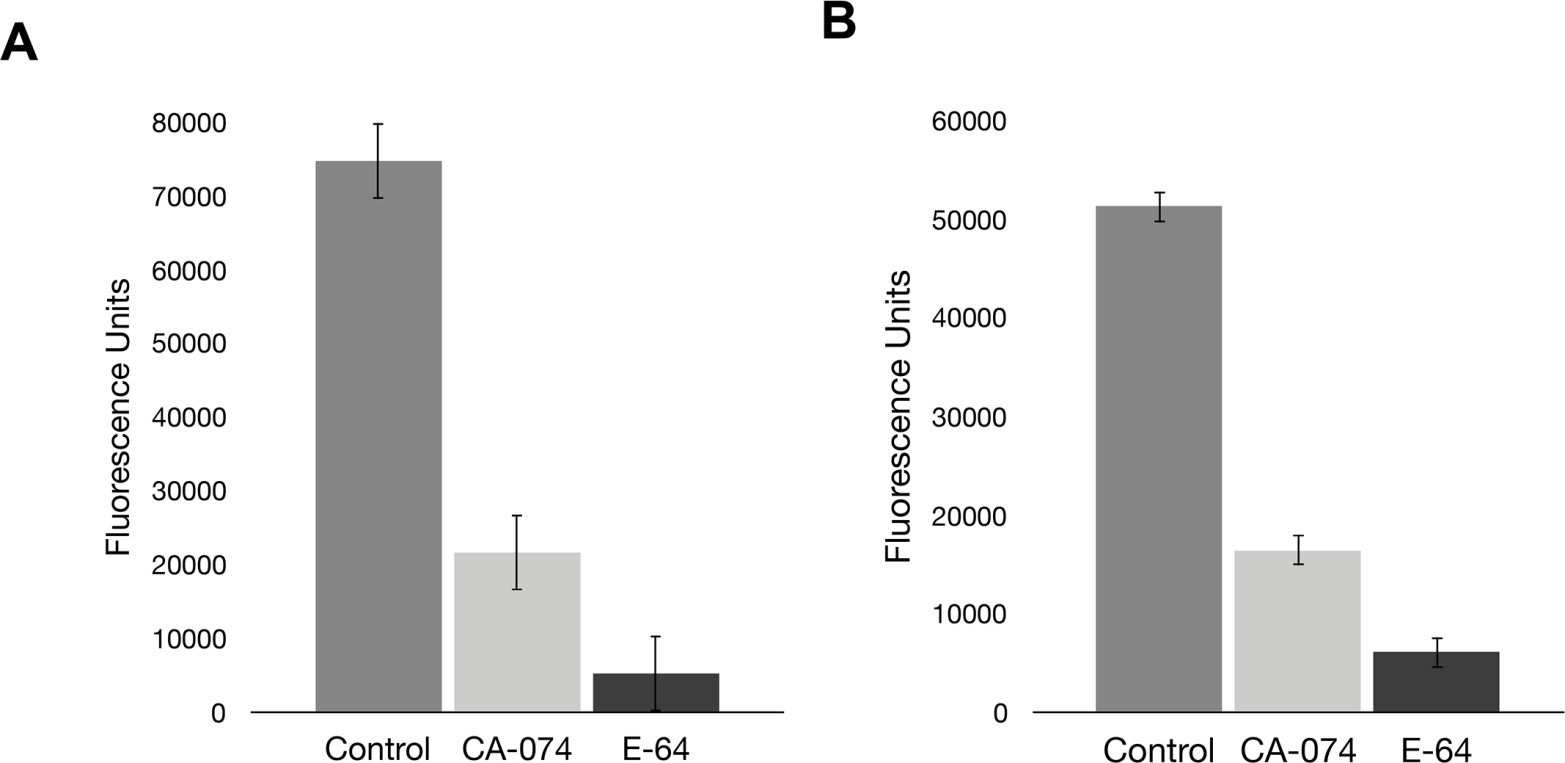
Total fluorescence of Z-RR-AMC and Z-FR-AMC cleavage by planaria lysate is reduced by inhibition of cathepsin activity. **(A)** Whole worm lysate was pre-incubated with 50μM E-64, a pan-cysteine protease inhibitor, before assaying with the fluorescent probe Z-RR-AMC. Pretreatment with E-64 reduced fluorescence by 93%. Inhibition of cathepsin B protease alone by CA-074 caused a 71% decrease in activity, indicating that the majority of proteolytic cleavage is caused by cathepsin B-like proteases. **(B)** Similar effects were observed when Z-FR-AMC, a probe cleaved by both cathepsin B and cathepsin L proteases, was used.

To examine the specific role(s) played by SmedCB, we used RNAi to assess whether loss of function was detrimental to worm viability and survival. Worms were first starved for two weeks before injection with ~100ng SmedCB dsRNA for three consecutive days. This protocol resulted in 80% reduction in SmedCB mRNA levels after fourteen days when compared to untreated worms (Fig 4A). This RNAi knockdown was specific to SmedCB and did not significantly impact mRNA levels of related proteases cathepsin L or cathepsin D (Fig 3B). Using the standard cathepsin B fluorescent substrate, z-Arg-Arg-*AMC* [40], protease activity was reduced in worm lysates by 81% fourteen days after SmedCB mRNA knock-down. (Fig 4B). Activity using this substrate was confirmed to be derived from cathepsin B because CA-074, a highly selective cathepsin B inhibitor, reduced total activity by 71% (Fig 5). In addition, protein hydrolysis using rhodamine-labeled albumin was reduced by 20% for 48 hours after feeding (Fig 4C). Taken together, these data confirm that SmedCB specifically plays a central role in protein digestion in *S*. *mediterranea*, much like cathepsin B1 does in *Schistosoma mansoni*.

### SmedCB is localized to the worm gut

*Schistosoma mansoni* expresses two isoforms of cathepsin B; SmCB1 is found in the gut and performs a digestive function, while SmCB2 is localized to the tegument where it serves an unknown purpose. We hypothesized that SmedCB would also be found in the gut of *S*. *mediterranea*, as indicated by its presence in the worm vomit, and would perform a role in digestion.

**Fig 6:**
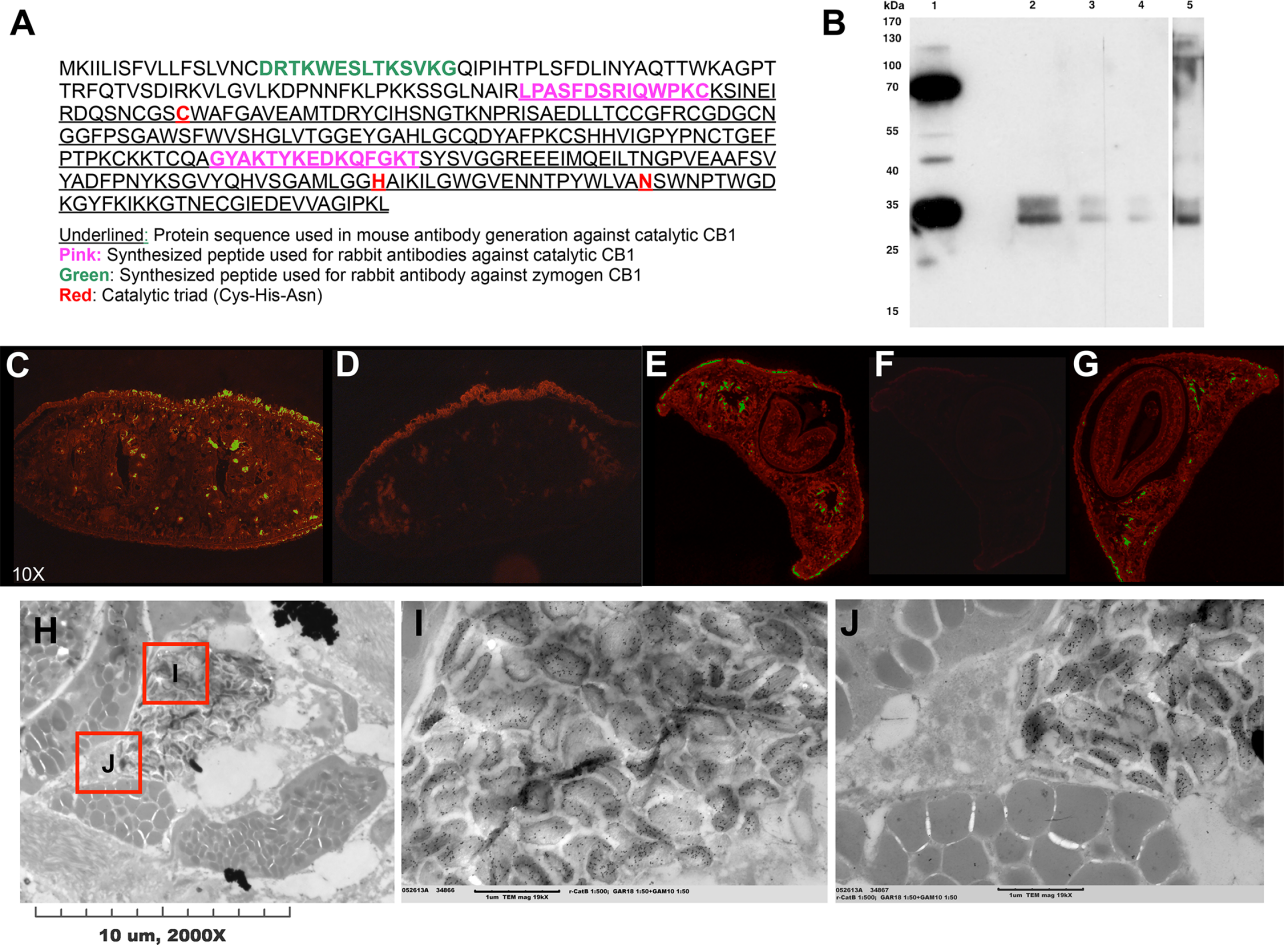
Immunofluorescence labeling and electron microscopy of zymogen and catalytic SmedCB shows strong labeling in cells lining the intestinal lumen. **A)** Several antibodies were generated against *Schmidtea* cathepsin B, including one against the catalytic region only (underlined region), and one against the zymogen tag and catalytic region (a combination of the green and pink peptides). **(B)** Western blot with antibody used to localize SmedCB at 1:5,000. Lane 1, recombinant prep of SmedCB from *E*. *coli*. Band at 70kDa is a dimer of SmedCB, lower band at 35kDa is zymogen SmedCB. Lanes 2–5 contain planaria lysate at 15μL, 7.5 μL, 5μL, and 15μL, respectively. Higher band (~37kDa) is zymogen, lower band (~27kDa) is catalytic domain. Lanes 1–4 use the mouse-derived antibody underlined in **A**, while lane 5 was blotted against the rabbit-derived antibody from the pink and green peptides in **A**. **(C)** Labeling of paraffin-embedded *S*. *mediterranea* cross sections of worms starved for one week with (1:100) anti-SmedCB zymogen pro-peptide antibody showed strongest signal in the cells lining the intestinal lumen. Comparison with the negative control **(D)** anti-rabbit secondary (1:100) only shows that the labeling on the border of the planaria cross sections is due to drying artifact. This pattern is also seen in worms with the anti-SmedCB catalytic domain only antibody (1:100) **(E)**. The negative control **(F)** with anti-mouse secondary only (1:100), shows labeling only on the edges of the cross sections due to drying artifact. Worms were also starved for two weeks prior to labeling **(G)** as opposed to one week in **C** and **E**. SmedCB labeling remained the same. **(H)** Cross sections of *S*. *mediterranea* worms (starved one week) were labeled with (1:100) anti-SmedCB zymogen pro-peptide antibody. Intestinal lumen seen as white space in the center of the image. **(I)** Immuno-gold labeling of antibody target, visualized as black dots, shows vesicle-like structures are heavily labeled. Other tissue, like the rounded, gray lipid droplets in **(J)** did not show labeling.

In order to determine the localization of SmedCB in *S*. *mediterranea*, we first needed to confirm that this protein was detectable in a whole worm lysate. Using the activity based probe DCG-04 [31], which specifically targets cysteine proteases, we observed a biotinylated band on an SDS-PAGE gel at 27 kDa. Mass spectrometry sequencing of the excised band confirmed that its identity as SmedCB (S2 Table). Knowing that SmedCB was present in whole worm lysate and not just in the regurgitation, we developed antibodies that could detect procathepsin B and mature cathepsin B. Peptides encoding a region of the propeptide, as well as the catalytic domain, were synthesized for antibody development (Fig 6A). Both antibodies specifically labeled SmedCB in the planaria lysate (Fig 6B). Immunohistochemistry using the zymogen SmedCB antibody labeled cells surrounding the intestinal lumen (Fig 6C and 6D). Localization of the catalytic region of SmedCB, found in both the full-length zymogen as well as the catalytically activated protein, was performed using an antibody generated against recombinant protein expressed in *Escherichia coli*. Immunohistochemistry showed that mouse antisera against the catalytic domain of SmedCB also labeled cellular vesicles surrounding the intestinal lumen (Fig 6E and 6F). Length of starvation did not affect localization of SmedCB (Fig 6G).

Previous histological analysis of planaria had identified two types of intestinal cells: “phagocytes” that absorb food for intracellular digestion, and secretory “goblet cells” that release digestive enzymes into the intestinal lumen [41, 42, 43]. Electron microscopy revealed that the proSmedCB localizes to vesicular structures within these intestinal cells (Fig 6H and 6I) but not neighboring cells, like lipid droplets (Fig 6J).

**Fig 7:**
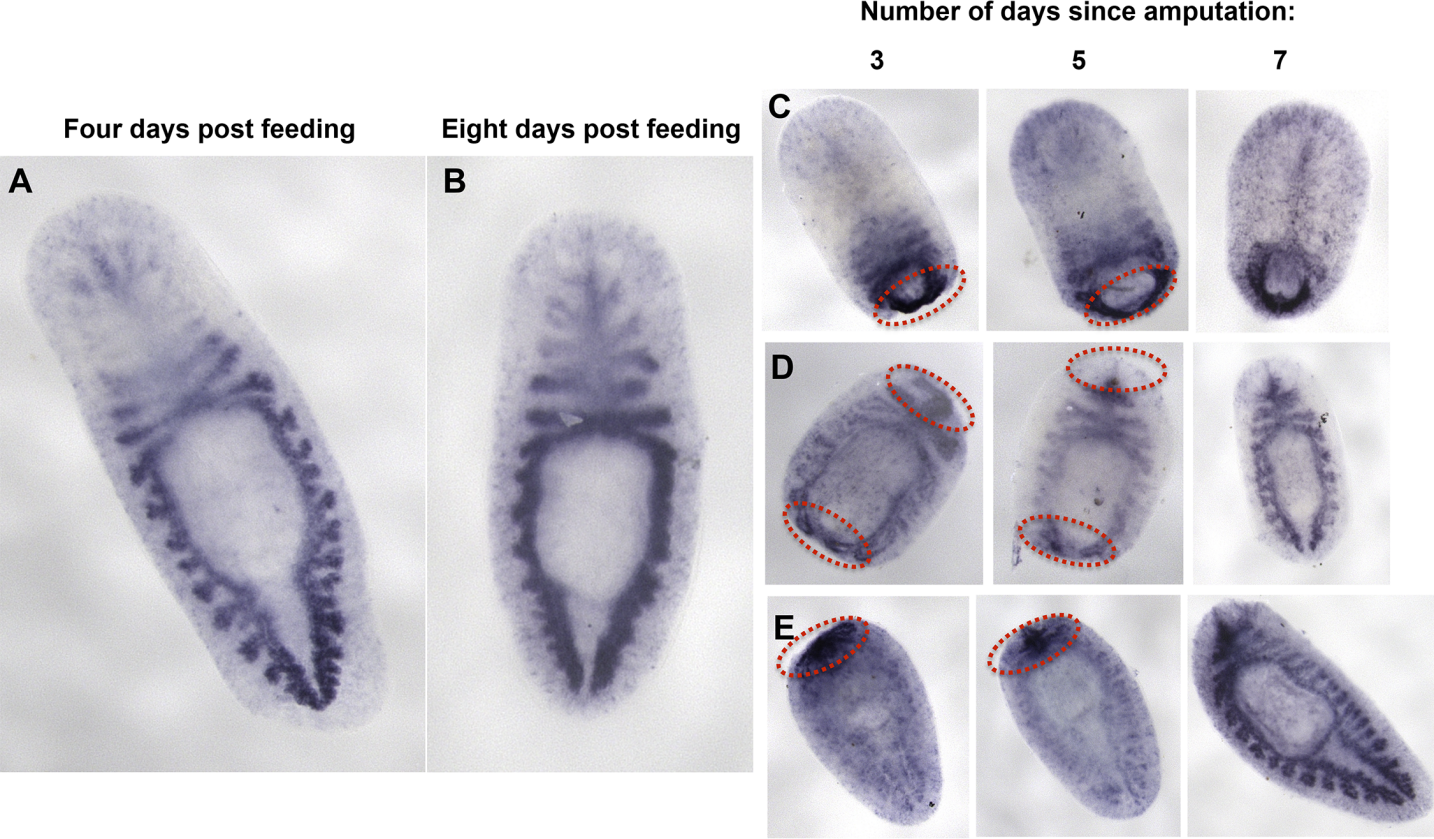
Whole-mount *in situ* hybridization of *SmedCB* shows labeling throughout the worm gut in intact and regenerating animals. An *in situ* DIG-12-UTP riboprobe against *SmedCB* shows labeling throughout the branched intestine. This pattern is observed in worms fixed four days after feeding **(A)** and eight days after feeding **(B**). For regenerating worms, animals were fixed three, five, or seven days after amputation. Some regenerating animals have strong labeling near the blastema (newly regenerated tissue, circled in red) as well as the newly-forming gut. Amputated heads **(C)** have labeling in the blastema up to seven days after amputation, while pharynxes **(D)** only show labeling near the blastema for three days. Tails **(E)** exhibit an intermediate phenotype with blastema labeled five days after amputation. All animals were treated with 1:100 dilution of the same riboprobe.

*SmedCB* localization was also confirmed via whole-mount *in situ* hybridization (WISH) to identify SmedCB RNA expression. The WISH protocol previously established [35], and modified [36], was used to maximize signal sensitivity. Planaria starved for four or eight days prior to sample preparation showed similar expression patterns despite the difference in worm feeding (Fig 7A and 7B). *SmedCB* is highly expressed throughout the branched intestine of the worm, confirming the labeling seen with immunohistochemistry. Furthermore, there appear to be punctae of SmedCB labeling in the mesenchyme, especially in the head where the gut signal is less pronounced. In regenerating worm fragments, labeling of the growing branched intestine as well as the punctae seen in intact worms is observed, although there are several differences in *SmedCB* expression. Planaria were cut into three segments: heads, tails, and pharynxes. Amputations were performed three, five, and seven days prior to treatment to assess the changes in *SmedCB* expression during regeneration. Head segments exhibit concentrated labeling near the blastema, the region of newly regenerated body tissue, throughout the time course. (Fig 7C). Regenerating pharynxes show some increased signal near the blastema, but this is only seen on worms that have been recently amputated. Worms that have healed over five and seven days no longer have an intense level of *SmedCB* near the blastema sites (Fig 7D). The blastema labeling in tail segments is observed at three and five days after amputation, but *SmedCB* appears to be localized to the same area as intact worms after a week of regeneration: the branched intestine (Fig 7E).

### Inhibition of SmedCB and cathepsin L slows *S*. *mediterranea* regeneration

**Fig 8:**
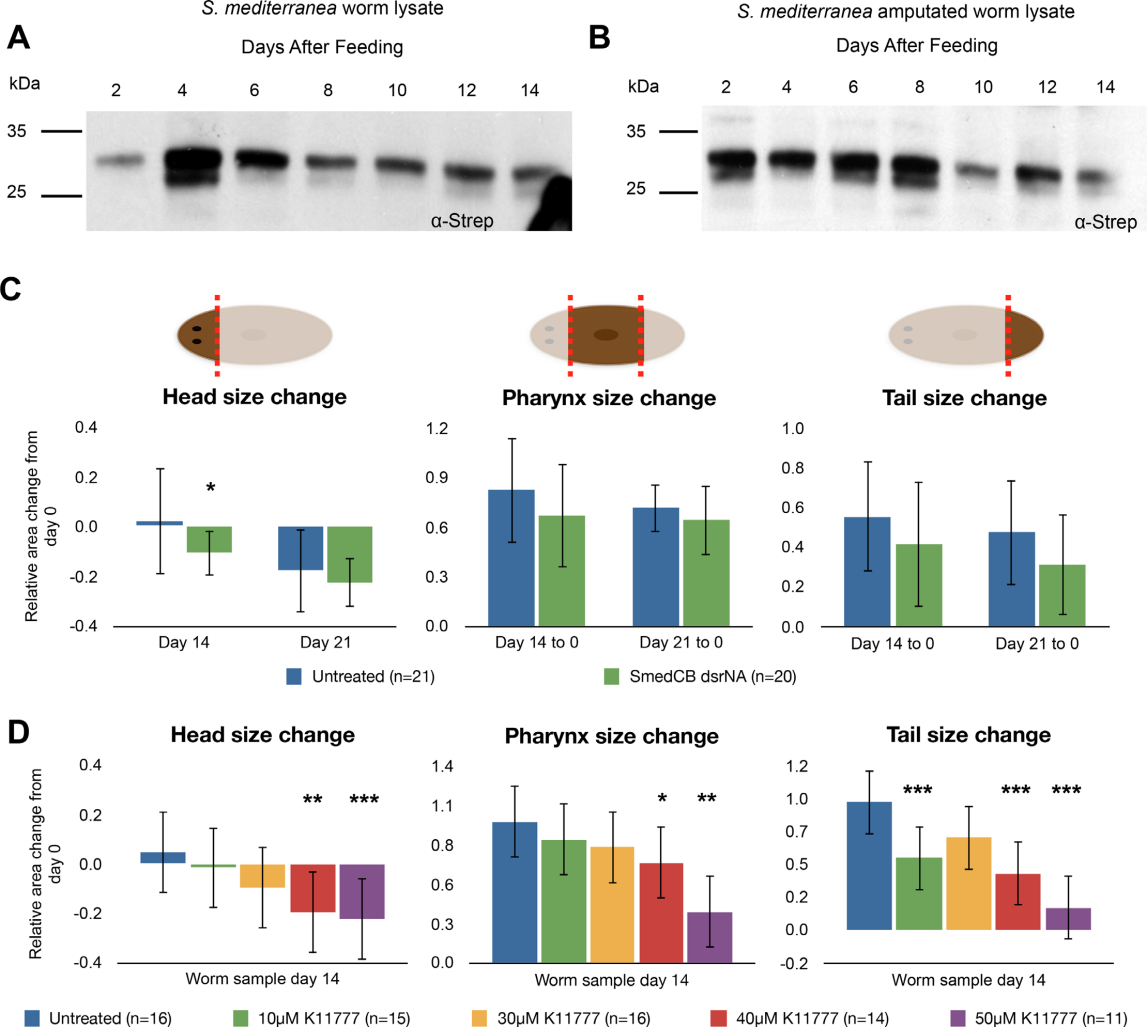
SmedCB shows a transient spike of protease activity following digestion and amputation, while chemical inhibition of both cathepsin B and L leads to a significant decrease in regeneration. **(A)** Whole worm lysate from *S*. *mediterranea* was labeled with the activity-based probe DCG-04 and resolved using SDS-PAGE. Western blot against the biotin tag of DCG-04 was used to analyze activity levels. *S*. *mediterranea* lysate showed an increase in cathepsin B labeling following digestion (day 4–6) before returning to base levels by day 14. **B)** When *S*. *mediterranea* worms were amputated following feeding, the increase in DCG-04 signal extended to day 8 before returning to baseline. The large upper band was quantified as SmedCB activity; five micrograms of total protein was used in each reaction with 10μM DCG-04. **(C)** Worms treated with SmedCB dsRNA did not show any significant defects in regeneration compared to untreated controls, but treatment with K11777 **(D)** showed a highly significant decrease in growth compared to vehicle worms. This effect was dose dependent. Relative changes in area are reported to account for any differences in initial size of worms selected. A negative value indicates that fragments shrank over time (heads), while positive values show relative growth. K11777 treated worms, on average, grew less (pharynxes, tails) and shrank more (heads), than untreated worms. These results were highly significant at 40 and 50μM treatment in all fragments, but not significant at lower concentrations.

Because of the interest in the regenerative ability of *S*. *mediterranea*, we also examined whether SmedCB might be required for regeneration as well as digestion. Another protease, transmembrane matrix-metalloproteinase A (Smed mt-mmpA), has been shown to modulate cell migration and delay new tissue growth [44]. Cathepsin B may modulate tissue growth as well given its localization to the regenerating blastema during *in situ* hybridization. We first tested whether SmedCB activity changed after feeding and amputation by incubating worm lysate with the activity-based probe DCG-04. This probe contains an electrophilic “warhead”, which becomes covalently attached to the nucleophilic residue of cysteine proteases. Specificity of the probe for various protease targets is achieved via a linker region between the warhead and a biotin tag, which is used to visualize protease labeling [32]. Following digestion, an increase in SmedCB labeling was observed for 2–6 days before returning to baseline during worm starvation, suggesting that cathepsin B was active when food was present, but inactive once digestion was finished (Fig 8A). Interestingly, a similar trend was observed when worms were amputated following digestion (Fig 8B). *S*. *mediterranea* worms take 7–8 days to fully regenerate; high SmedCB activity during this period suggests a possible use for cathepsin B during worm growth and regeneration.

SmedCB RNAi treated worms did not show major defects in regeneration, although regenerating worm segments did grow less than untreated worms (Fig 8C). This was quantified through the relative size increase of regenerating worms. Relative size increase was measured comparing the change between day 14 and day 0 worms divided by initial size to normalize for any variation in the size of selected animals. This trend of decreased growth in RNAi worms was consistent over two and three weeks, but it was not statistically significant. Therefore, despite the change in active protease levels observed in regenerating worm lysates, SmedCB alone does not appear to play an essential role in growth rates of *S*. *mediterranea* during regeneration.

We next examined whether inhibition of cathepsin B and L in tandem had any effect on regeneration and found that in contrast to RNAi of SmedCB alone, chemical inhibition of both proteases greatly reduced growth of treated worm fragments (Fig 8D). This effect was dose dependent. In general, head fragments have fewer neoblasts than pharynxes and tails [45]. Therefore, even vehicle treated heads do not grow much larger than their original size after amputation. Untreated heads had a relative size increase of only 0.045, as compared to 1.0 for pharynxes and 0.93 for tails. This means that while pharynxes and tails increased their size by almost 100% over two weeks of regeneration, heads tended to remain the same size. K11777 treated worms had significant reductions in growth and, in the case of heads, increased shrinking. Heads treated with 40μM K11777 were almost 20% smaller than when they were amputated. Pharynx and tail relative size increase stood at 0.72 and 0.41, respectively. Compared to vehicle treated worms, all three regions of worms had a significantly lower relative size increase when treated with K11777. These data suggest that while knockdown of SmedCB alone is not enough to hinder to regeneration, chemical inhibition of SmedCB and cathepsin L proteases inhibits planaria ability to grow following amputation.

## Discussion

Proteases perform many vital functions in both free-living and parasitic flatworms. For free-living flatworms like *Schmidtea mediterranea*, effective protein digestion is essential to growth and reproduction. Maturity of asexual planarians depends on size, which directly correlates with feeding; the more an animal is fed, the more it will divide and reproduce [46]. The major digestive enzymes in the family *Platyhelminthes* are cysteine proteases, in contrast to vertebrates, which predominantly use serine proteases. Several proteases often function as a network for protein digestion. The parasitic worm *Schistosoma mansoni* uses a proteolytic cascade of cysteine and aspartyl proteases to degrade host albumin and hemoglobin into amino acids. *S*. *mansoni* relies on the cysteine protease cathepsin B for digestion of albumin, although cathepsin L, legumain, and the aspartic protease cathepsin D are involved in later steps of albumin processing [10]. In contrast to the role of cathepsins B and L in albumin degradation, cathepsin D plays the primary role in hemoglobin digestion by schistosomes [47]. Interestingly, while homologs of cathepsin B, L, and D were found in free-living *Schmidtea* worms, no homolog for legumain was identified (Table 2). It is therefore presumed that legumain performs a “parasitic” function, although its precise role in the host-parasite relationship is unknown [48]. Legumain has been implicated in the digestion of host hemoglobin in flatworm parasites [10, 49] as well as other blood-feeding ecto-parasites, like ticks [50].

We were able to induce regurgitation in *S*. *mediterranea* to examine the major proteases present and active in the flatworm gut lumen. We used peptide substrates and class specific protease inhibitors to determine that aspartyl, cysteine, and metalloproteases were present and active in the worm regurgitant. Treatment of live worms with a combination of aspartyl and a cysteine protease inhibitor reduced protein degradation in the gut by 46%. While the physiological pH of the *S*. *mediterranea* gut is unknown, *S*. *mansoni* regurgitant has been estimated to be pH 6.0–6.8 [20] while the *C*. *elegans* gut pH varies from pH 3.6 to pH 6.0 depending on specific location [51]. Previous work found that optimal degradation of albumin and hemoglobin by *S*. *mansoni* proteases, occurred at pH 4.0 and not pH 6.0 [10]. Pepsin-type aspartyl proteases generally have little or no activity above pH 5.5 and therefore the pH of *S*. *mediterranea* is more acidic than pH 5.5 since pepstatin has such a profound effect on albumin degradation. In support of this, the number of cleavages sites generated by proteases active at pH 3.5 and 5.5 were considerably greater than at pH 7.5. In fact, only metalloproteases with exo-peptidase activity were detected a neutral pH and these enzymes are likely to play a role in generating single amino acids from peptide termini in the later stages of degradation. Taken together, we hypothesize that protein degradation is initiated at pH 5.5 or lower by a combination of aspartyl and cysteine proteases and these peptides are further processed by exo-peptidases in a region of the gut where the pH is closer to neutral.

Feeding assays with *S*. *mediterranea* confirmed the importance of cysteine and aspartyl proteases *in vivo*. Knockdown of cathepsin B activity through RNAi significantly decreased protein digestion in worms. This mirrors the function of SmCB1 in *S*. *mansoni*. Concurrent inhibition of SmedCB and cathepsin L activity using the inhibitor K11777 resulted in a further decrease in digestive ability. This implies some redundancy in the activities of cathepsin B and L. When SmedCB is specifically knocked down with RNAi, cathepsin L can compensate for some of the loss in digestive ability. However, when both proteases were chemically inhibited, digestion of rhodamine-labeled albumin was profoundly decreased. Aspartyl proteases also act in the process of digestion in *S*. *mediterranea*. Although no aspartyl proteases were detected in the worm regurgitant by mass spectrometry, it is possible that cathepsin D is not secreted directly into the intestinal lumen. Previous work in *S*. *japonicum* found that cathepsin D localized in digestive vacuolar compartments lining the gastrodermis, where it aids in the breakdown of host hemoglobin [52, 47]. The primary site of action of cathepsin D in these worms is thought to be in the gastrodermal lysosome or endosome. Blocking the action of aspartyl proteases with pepstatin resulted in a significant decrease in digestion in live *S*. *mediterranea*, and concurrent inhibition of cysteine and aspartyl proteases saw an almost complete loss of digestive ability. This suggests that initial digestion might take place in the intestinal lumen via the action of cysteine proteases and the remaining fragments are taken up into cells where further degradation by aspartyl proteases occurs. Inhibition of both cysteine and aspartyl proteases showed a dramatic decrease in digestive ability, suggesting that both of these classes of proteases are critical for proper digestion in the worm gut. Inhibition of metalloproteases had no effect on digestive ability, suggesting that the metalloproteases detected in the worm regurgitant are not involved directly in the breakdown of food.

*SmedCB* RNA expression was visible throughout the branched intestine. This suggests that SmedCB transcription occurs throughout the gut epithelium followed by packaging of the translated protease in adjacent intestinal cells. This is confirmed by antibody localization of translated protein in secretory vesicles of intestinal cells. This suggests that SmedCB performs a similar role to SmCB1 in *S*. *mansoni* and that vesicles are used to store full-length SmedCB before cleavage of the zymogen form and secretion into the intestinal lumen. In regenerating worms, *SmedCB* is expressed in the newly formed gut as well as near the blastema during early stages of growth. It is unclear whether this expression is due solely to the formation of the gut near the blastema, or if SmedCB plays other important roles during tissue remodeling, requiring its increased presence and activity at the site of regeneration.

While SmedCB alone does not impact the ability of *S*. *mediterranea* worms to regenerate, inhibiting the activity of both cathepsin B and L did result in a significant decrease in growth rate. During regeneration, old structures are broken down through both autophagy and apoptosis [53]. Apoptosis occurs in two waves: an initial localized response near the wound site and followed by a systemic response. Even preexisting tissues undergo apoptosis in order to maintain their correct proportions within the worm [54]. Perhaps cysteine proteases are involved in tissue remodeling and turnover during regeneration and their absence slows the rate of this process. The lack of severe morphological effects during regeneration in cathepsin B and L inhibited worms suggests that while these proteases may play a small role in growth, they are not entirely essential to worm survival.

We have exploited the ease of use of *Schmidtea* to confirm the conservation of a major cysteine protease in free-living and parasitic flatworms. We have found that not only is cathepsin B involved in digestion in free-living worms, but it also plays a role in growth and regeneration. Schistosome parasites also have neoblast-like stem cells, so there may be other aspects of worm growth or life cycle alterations that are conserved between planarians and *Schistosoma* [7].

## Supporting Information

S1 Video Vomiting is induced in *Schmidtea mediterranea* by the addition of 3% ethanol. Worms placed in 3% ethanol undergo muscular contractions causing them to regurgitate gut contents. This 15X time lapse shows the appearance of partially digested beef liver (white solid) from the worm pharynx. Worm vomit collected for this study was from worms which had been starved for one week; no solid food was collected. Worm vomit was concentrated and filtered before use. Worms were returned to worm water to recover.
(M4V)

S1 Table Proteins identified in *Schmidtea* regurgitant. Samples of worm regurgitant were collected, concentrated, and trypsinized for peptide sequencing by mass spectrometry. Thirty proteins were found in all three replicates, thirty-two in at least two replicates, and sixty proteins were found in only one replicate. Proteins are listed by their intensity sums detected via mass spectrometry.
(XLS)

S2 Table 27kDa band labeled by DCG-04 is SmedCB. Whole worm lysate was labeled with the activity-based probe DCG-04 and separated via SDS-PAGE. A 27kDa labeled band was excised, tryspinized, and sequenced via mass spectrometry. Samples of worm regurgitant were collected, concentrated, and trypsinized for mass spectrometry.
(XLSX)
